# Areas of enduring COVID-19 prevalence: drivers of prevalence and mitigating strategies

**DOI:** 10.1186/s12889-023-15723-7

**Published:** 2023-06-21

**Authors:** Catherine Lewis, Sheena Johnson, Angelique Hartwig, Janet Ubido, Anna Coleman, Nicola Gartland, Atiya Kamal, Amit Gaokar, Christopher J. Armitage, David Fishwick, Martie van Tongeren

**Affiliations:** 1 Division of Population Health, Health Services Research & Primary Care, University of Manchester, Manchester, England; 2 Alliance Manchester Business School, University of Manchester, Manchester, England; 3 Birmingham City University, Birmingham, England; 4 Rochdale Borough Council, Manchester, England; 5 Manchester Centre for Health Psychology, University of Manchester, Manchester, England; 6 Health and Safety Executive (HSE), Liverpool, England

**Keywords:** Community engagement, COVID-19, Deprivation, Employment, Health inequalities, Partnership working

## Abstract

**Background:**

UK local authorities that experienced sustained high levels of COVID-19 between 1st March 2020 and 28th February 2021 were described by the UK Scientific Advisory Group for Emergencies as areas of enduring prevalence. This research was carried out in order to examine the views of local authority Directors of Public Health, who played a crucial role in the local response to COVID-19, on reasons for sustained high levels of prevalence in some areas, alongside an investigation of the mitigation strategies that they implemented during the course of the pandemic.

**Methods:**

Interviews were conducted with Directors of Public Health in 19 local authority areas across England, between July and November 2021. This included nine areas identified as areas of enduring prevalence and ten ‘comparison’ areas.

**Results:**

The outcomes of this study suggests that the geographical differences in prevalence rates are strongly influenced by health inequalities. Structural factors including deprivation, employment, and housing, due to their disproportionate impact on specific groups, converged with demographic factors, including ethnicity and age, and vaccination rates, and were identified as the main drivers of enduring prevalence. There are key differences in these drivers both within and, to a lesser extent, between local authorities. Other than these structural barriers, no major differences in facilitators or barriers to COVID-19 mitigation were identified between areas of varying prevalence. The main features of successful mitigation strategies were a locally tailored approach and partnership working involving local authority departments working with local health, community, voluntary and business organisations.

**Conclusions:**

This study is the first to add the voices of Directors of Public Health, who played a crucial role in the local COVID-19 response. Areas of enduring prevalence existed during the pandemic which were caused by a complex mix of structural factors related to inequalities. Participants advised that more research is needed on the effectiveness of mitigation strategies and other measures to reduce the impact of structural inequalities, to better understand the factors that drive prevalence. This would include an assessment of how these factors combine to predict transmission and how this varies between different areas.

**Supplementary Information:**

The online version contains supplementary material available at 10.1186/s12889-023-15723-7.

## Background

Since the start of the SARS-CoV-2 pandemic in March 2020, there have been regional variations in community transmission rates throughout the United Kingdom (UK) [1]. Epicentres during the pandemic have shifted from Greater London to the Midlands and the North of England [2]. However, data have demonstrated that there are also regions which have observed enduring increased rates of SARS-CoV-2 prevalence within England.

Local authorities in England that experienced sustained high levels of COVID-19 were described by the UK Scientific Advisory Group for Emergencies (SAGE) Regional Variations sub-group as ‘areas of enduring prevalence’ (AEP) [3]. SAGE identified the ten local authorities with the highest number of days spent in the epidemic phase, defined as those with the highest mean number of daily cases, highest variability, and the strongest correlation between case numbers across consecutive days, between 1/3/20 and 28/2/21 [4]. SAGE’S definition of enduring prevalence was used to carry out this study.

SAGE highlighted that factors related to employment, including income and ability to work from home, might be linked to COVID-19 prevalence because they have an impact on workers’ ability to self-isolate if needed [3]. This aligns with the findings of wider research [5] which also identified differences in mortality rates according to occupation, observing that COVID-19 mortality rates were highest among those working as taxi and cab drivers or chauffeurs followed by those working in other ‘elementary’ occupations such as cleaners or catering assistants, and as care workers and home carers. Previous research [6] also suggested that COVID-19 vaccination rates were higher among people working in professional and managerial occupations than people working as general labourers, packers, or cleaners.

SAGE reported higher prevalence rates among certain groups, including people from minority ethnic groups, and people living in poor quality or overcrowded housing or in multigenerational households [7], in alignment with wider research [8].

In terms of mitigating strategies used by local authorities to control COVID-19 prevalence, there are many examples of good practice. The Local Government Association (LGA) [9] has published case studies of good council practice that include vaccination, testing strategies, and implementation of local test and trace systems. The King’s Fund [10] also explored the roles of Directors of Public Health (DsPH) during the pandemic and concluded that they had been instrumental in the local public health response to COVID-19.

However, there has been no published literature to date exploring regional variations of AEP and comparison areas (CA). The current research addresses this important issue and will help to inform the establishment of support mechanisms that will help minimise regional disparities in transmission rates. In addition, the current study is the first published research to include the voices and experiences of DsPH on COVID-19 transmission. It is vital to learn from the experiences of DsPH as they are key decision makers who are responsible for setting public health objectives in their local authority areas, and as described above they played a pivotal role in the local public health response to COVID-19 [10].

The aims of the current research were to gain expert views and insight from local authority DsPH into what might be the main factors that cause regional disparities in COVID-19 infections and better understand why certain places appear to have consistently relatively high prevalence of COVID-19 infections compared to other areas. This was done by comparing the AEP defined by SAGE, to areas that were similar to them, for example in terms of geographical location, deprivation, or population mix, but were not defined by SAGE as AEP. This research also aimed to examine the perceived impact of mitigation strategies used by DsPH on variations in COVID-19 transmission rates, along with barriers to implementing mitigating strategies.

## Methods

DsPH in the ten local authorities identified as AEP by SAGE [3] were invited to take part in the research, and nine DsPH agreed. A set of ten CA were selected using purposive sampling, according to recommendations by DsPH, the Association of Directors of Public Health (ADPH) and Public Health England (PHE) (now the UK Health Security Agency (UKHSA). Recommendations were made based on statistical similarities and similar demographic factors including deprivation or age, to the AEP, but with lower COVID-19 prevalence. Two of the CA were included as they had been identified as statistical neighbours to two of the AEP [11]. Statistical neighbours are defined as those that are similar in terms of levels of deprivation, whether urban or rural, and on population mix of young, old, and ethnic minorities [11]. An analysis of the differences in infection rates between the AEP and CA during the time period that SAGE used to identify AEPs, 1st March 2020 to 28th February 2021, was also conducted as part of the current research.

As part of a wider project conducted by the authors of this study [12], data on key indicators, including deprivation levels, vaccination rates, and factors related to employment, were inspected for all the local authorities that were included in the study. An overview of the findings of this wider study is presented at the beginning of the results section.

Prior to commencing the research, the research team used the University of Manchester’s ethics decision tool which confirmed no ethical approval was required for this study. The University of Manchester does not require formal ethical review for interviews where the subject matter is limited to topics that are strictly within the professional competence of the participants. Standard ethical procedures were followed, and informed consent was obtained from all subjects.

A steering group was established in order to oversee the project. The steering group included external partners including the Health and Safety Executive (HSE), UKHSA and local authority and university partners, as well as internal partners.

Semi-structured interviews were carried out with 18 DsPH and one other senior lead in 19 local authorities across England, between July and November 2021. Nine interviews were in AEP and ten were in CA, to gain in-depth understanding of why certain areas experience sustained prevalence. Local authorities were anonymised when reporting results. Of the nine AEP, three were in the North West, two were in Yorkshire and Humber, two were in the Midlands, and two were in the South East. Three of the ten CA were in the North West, one was in Yorkshire and Humber, two were in the Midlands, two were in Greater London, one was in the South East, and one was in the South West.

Interviews lasted approximately one hour and included 15 questions. Participants were asked: what they felt were the drivers of high, sustained levels of prevalence; what were the mitigating strategies that were implemented nationally and locally during the pandemic; and to identify barriers to the implementation of these strategies. The interview schedule was devised based on existing literature, including previous SAGE reports [3, 7] as well as wider research [5, 6] and in collaboration with the project steering group, with PHE (now UKHSA) and with the ADPH. Interviews were conducted online via Zoom or Teams by two researchers. The interview schedule is included in Appendix 1.

Interviews were transcribed and thematically analysed using an iterative coding process [13]. Two researchers coded the interviews using NVivo, of which a third were second reviewed by the wider research team in order to mitigate the risk of the introduction of bias by the researchers during the coding process. Development of the initial coding framework was guided by the research questions and topics that were raised by participants during the interviews. The codes were iteratively adapted and restructured throughout the initial coding stage and as a result of discussions between the researchers.

To begin with, all transcripts were coded using the initial coding framework. As DsPH were asked to discuss factors that are likely to drive enduring prevalence in their local authority area and local strategies that may be effective in reducing COVID-19 rates, responses were analysed separately before comparing AEP and CAs. The aim of the within- and between-group comparison was to identify similarities and differences between areas of high prevalence and low prevalence. The comparison was conducted firstly by compiling instances discussed by DsPH with regards to factors contributing to enduring prevalence, effective local and national strategies to manage prevalence rates and the main barriers in managing COVID-19 locally and nationally. This was followed by an examination of participants’ perceptions of how their local authority area compared to other local authorities with similar characteristics in terms of these drivers of prevalence. Participants’ perceptions of the impact of the drivers on transmission rates in the local authority area, and their experiences of the interactions between the ‘risk’ factors that they described were also examined. Similarly, participants’ perceptions of the effectiveness of the mitigating strategies that they had used to control COVID-19 transmission, along with barriers and facilitators to the implementation were compared.

Finally, one-to-one comparison of those AEP and CA that are statistical neighbours was conducted. This analysis provided an opportunity for deeper exploration of reasons for differences in prevalence rates in similar areas, along with an exploration of DsPH opinions on the impact of mitigating strategies. Quotes have been used to illustrate points in the text. Each local authority was assigned a unique identifier (e.g. P1, CA) which indicated whether the participant was from an AEP or a CA.

## Results

This section begins with an overview of differences in infection rates between the AEP and the CA, followed by a brief overview of differences in key indicators between the AEP, CA and the national average that were identified in a wider study that was conducted by the authors of the current study [12]. The drivers of enduring prevalence that were identified by DsPH are also presented in this section, along with mitigating strategies that DsPH used during the course of the pandemic.

### Differences in infection rates between AEP and CA

SAGE [4] identified AEPs using the time period from 1st March 2020 to 28th February 2021. Inspection of infection rates during this time period suggests that, although there were no significant differences between the AEP and CA during this time period as a whole, infection rates in the AEP were 48% higher than in the CA between 1st April and 30th September 2020. During the time period from 1st October 2020 to 28th February 2021, when infection rates across England were higher [14], there was no clear difference in infection rates between the AEP and the CA.

### Overview of differences in key indicators between the AEP and CA

As part of a wider study [12], data on key indicators, including deprivation levels, vaccination rates, and factors related to employment, was inspected for all the local authorities that were included in the study. Indicators gathered were based on the most recent statistics that were available at the time of data collection in Autumn 2021. As shown in Table 1 below, differences between the AEP overall and CA overall were analysed for each key indicator and were compared to the national (England) average. Median values for AEP and CA were calculated and compared using Mann Whitney tests to identify any significant differences.

Overall, there were higher levels of deprivation in AEP than in CA and the national average. The proportion of people aged over 16 from ethnic minority groups was higher in the AEP (21.4%) than in the CA (14.3%) and the national average (13.6%). The age profile in the AEP was younger, with less people aged over 65 (15.5%) in the AEP compared to the CA (17.0%) and the national average (18.4%).

Population density (population per square kilometre) was much higher overall in both the AEP (1,498) and CA (2,022) than the national average (432). The percentage of people in overcrowded housing was higher overall in the AEP (6.0%) than in the CA (3.4%) and the national average (4.8%). The proportion of the population with a second COVID-19 vaccination was generally much lower in AEP (66.3% compared to 74.7% for CA, and 80.1% for the national average. Data on booster uptake was not available at the time of data collection.

The percentage of people in employment was significantly lower in AEP (69.7%) than in CA (75.4%) and the national average (75.1%). The percentage of people working in occupation group 8–9 (process, plant and machine operatives, and elementary occupations) was significantly higher in the AEP (20.7%) than in the CA (15.9%) and the national average (14.7%).

**Table 1:** Table to show key indicators for AEP, CA and England average

Indicator	AEP Median (middle) values * = AEP significantly different to comparison area (p < 0.05, Mann-Whitney test)	CA Median (middle) values	England Average (#=Great Britain)
Deprivation Socioeconomic deprivation decile group, from 1 (high) to 10 (low), IMD 2019	3.0	4.5	5.5
Ethnicity % population aged 16 + from ethnic minorities 2016	21.4%	14.3%	13.6%
% Age 65+ 2019	15.5%	17.0%	18.4%
Population density total population per square kilometre (2019)	1,498	2,022	432
% In Overcrowded housing 2011	6.0%	3.4%	4.8%
% in Employment 2020/21	69.7%*	75.4%	75.1%
Employment by occupation, % Group 8–9, 2020/21 Process, plant, and machine operatives. Elementary occupations	20.7%*	15.9%	14.7%^#^
% vaccinated with 2nd dose (as at 24/11/21)	66.3%	74.7%	80.1%

*Sources: PHE and NOMIS; Reproduced from Lewis et al., 2022.*

### Drivers of prevalence

DsPH associated higher, prolonged COVID-19 prevalence rates with deprivation, population density, overcrowded housing, work-related factors including the nature of work and employment conditions, and vaccination uptake. These factors often intersected with demographic factors including ethnicity and age. Deprivation and nature of work was often jointly discussed as creating barriers for people to financially afford to self-isolate or to work from home. Overcrowded housing conditions and densely populated areas were mentioned as facilitating rapid transmission within communities. Participants emphasised that the interaction of various factors could create a “perfect storm” for enduring high transmission rates.

### Deprivation

All participants identified factors linked to deprivation as one of the most important influences on enduring prevalence, particularly in terms of the proportion of people on low incomes or in low quality or overcrowded housing. Participants in both AEP and CA highlighted that wards, super-output areas or geographical areas within their local authorities were more deprived than others: with some participants saying that they could have predicted where the areas of enduring prevalence were likely to be based on deprivation:

*“I mean, it’s classic public health, it’s what we’re seeing, you know, you can pinpoint the areas where you’re going to end up with higher prevalence.”* (P1, CA)

Although all participants discussed the impacts of deprivation, participants in the AEP were more likely to describe the local authority as having higher levels of deprivation than the national average or than neighbouring local authorities, and they reported that these higher deprivation levels may have caused increased transmission levels. Participants also highlighted the impacts of deprivation when combined with other factors such as overcrowded housing or with demographic factors such as ethnicity, as discussed in the sections on housing and demographic factors below.

### Employment

All participants identified factors relating to employment as one of the drivers of enduring prevalence. Participants linked factors including being unable to work from home and therefore coming into contact with a greater number of people to increased risk of transmission. Participants said that people who did not receive sick pay, who were on zero-hours contracts, were in low-income jobs, had precarious employment terms, or were self-employed but did not qualify for support grants, found it more difficult to take time off work in order to self-isolate. This may have also made them more reluctant to take a COVID-19 test, which participants suggested could have had an impact on prevalence rates:

“*So, I think we had quite a lot of people, where isolation was difficult financially, in terms of zero hours contracts and not getting paid holiday and sick time and all of those issues, which I guess, led to some reticence to get tested*.” (P3, CA)

Several participants said that employers’ attitudes to sickness absence also influenced employees’ ability to self-isolate when necessary, as some workers might have felt that their jobs were at risk if they took time off work to self-isolate. Some also discussed disparities between workplace policy and practice:

*“We’ve got more people who are in insecure work or potentially in jobs where, you know, employment practices aren’t gold standard. So, people will feel their jobs are at risk if they can’t go in, which then, obviously, means that there’s pressure for people not to self-isolate or not to test if there’s that risk that they could lose their job.* ” (P6, AEP)

Participants also linked sustained prevalence to factors related to employment, including workers living in shared accommodation and car sharing, especially at the beginning of the pandemic when people were advised to avoid using public transport. Some reported that car sharing was more common among workers who were on lower incomes trying to reduce transport costs, those who travelled short distances to work, or worked in factories that were difficult to access via public transport. Participants also said that use of public transport increased transmission risk, especially when public transport was overcrowded.

There were many references to ability to self-isolate from participants in both AEP and CA, although participants from AEP made slightly more references to this. Participants in the AEP were more likely to suggest that this issue affected a higher proportion of their residents than the national average, or a greater proportion than in neighbouring local authorities. Participants linked this with deprivation, and those in CA that had significantly higher levels of deprivation than the national average were also likely to discuss this issue. Additionally, participants in the AEP, and participants from CA that were more deprived than the national average, made slightly more references to car sharing than other participants, and highlighted that this issue was more likely to affect their residents than residents living in neighbouring local authorities:

“*Work patterns…so it’s not people that are travelling huge distances, they travel fairly tight distances, probably shared transport, because they’re not affluent enough to have lots of cars that sit around and you can isolate yourself, and then the nature of the work might be in quite confined industries…And it was a common…. theme of the factors, they often live together, socialise together, and travel to work together*. ” (P15, AEP)

### Housing

Most participants identified issues related to housing as key drivers of prevalence. Household transmission of COVID-19 was reported to be more likely in larger households, with variation in transmission rates from different variants of COVID-19. Several participants said that the risk of household transmission was increased for people living in houses of multiple occupancy, or in three generational households where younger people might contract COVID-19 and it would then spread to older family members. Overcrowding made it more difficult for people to isolate from other members of their household where necessary. Several participants also said that household transmission rates were higher in certain geographical areas within their local authority than others:

“*We have a significant number of houses of multiple occupancy within the town, but actually we’ve got a significant probably unspoken number of houses with multigenerational families, so very large families living within single households. What we have identified with COVID is very much that if COVID gets into the house then you are very likely to pass it throughout your close contacts within that house*. ” (P11, CA)

Participants in the AEP were more likely to identify overcrowded housing as a risk factor for prevalence. Participants in AEP were more likely to report that their local authority area had higher than average levels of overcrowded housing, therefore a higher proportion of residents were at greater risk because of this issue. Conversely, one CA participant reported that housing in the local authority area was of high quality and was highly regulated by the local authority. The participant suggested that this, along with the implementation of comprehensive initiatives to support people who were homeless during the pandemic, might be one reason why the area had experienced lower transmission rates than neighbouring local authorities that had been defined as AEP, despite the local authority having higher than average levels of deprivation:

“*We have very good housing standards in XX… we do have private rented stock, but not to the same extent. So, that has, you know, enabled us to really focus on healthy housing. So … it’s a very much public health, person centred approach to housing policy*. ” (P7, CA)

In addition, although national guidance on reducing transmission was often based on the idea of a ‘household’ being limited to people living in one house, participants said that residents sometimes viewed their ‘household’ as spanning much further than this, often in terms of caring responsibilities, including child-care/ provision of food, increasing the risk of community transmission. Participants said that this was linked to deprivation and happened when people relied more on family and/or other close social contacts than on more formal networks. Participants in AEP were more likely to identify this as a risk factor than those in CA, or to suggest that it had an impact on a larger proportion of their residents than average, because of high deprivation levels in the local authority area:

“*Even within a street you could almost follow the epidemiology of not just communicating it between each other… they see those other households, even though they’re at different addresses, as part of their household…. You know, they had… caring responsibilities and it was just the way that they lived, they just happened to have slightly different front doors, but they were all one family or one household as far as they were concerned.* ” (P8, AEP)

### Population density

Several participants across areas of varying prevalence identified population density as a risk factor for community transmission, although no meaningful differences were identified between AEPs and CAs.

*“In our county, it’s the areas of denser population, it’s the areas of poorer people........ where people are having to go out to work. And that absolutely matches definitions of populations impacted by enduring transmission.” (P12, CA)*

### Mobility in and out of local authority areas

Most participants, but especially those in CAs, discussed the impact of travel in and out of their local authority area on prevalence, including travel by tourists, university students and commuters. This affected transmission to a greater extent when neighbouring local authorities had high transmission rates, which was often linked to deprivation in these neighbouring areas. Several participants discussed the impact of people travelling into the local authority for reasons including shopping and visiting hospitality venues such as pubs and restaurants, either due to lockdowns in other areas or because they had family or other connections there.

*“One of the reasons that we tipped from a (tier) two into a four in a matter of two weeks, is because it was coming up to Christmas and nobody could shop in London….so they all just drove into XX ‘cause you could go shopping there*. ” (P8, AEP)

### Vaccination rates

Most participants discussed inequalities in vaccine uptake although they asserted that these inequalities were alleviated to some extent by the wide range of interventions and initiatives that were implemented to tackle this. Many participants said that people in more deprived areas within the local authority were less likely to take up the offer of a vaccine, reflecting patterns of uptake of vaccines for other illnesses, for example the influenza vaccine [15]. Participants in AEP were more likely to report that vaccination rates were lower than the national average than those in CA. This was confirmed by the key indicators detailed in the wider study [12] (Table 1). Several participants said that one potential explanation for this was those residents from more deprived populations, particularly, might feel mistrust towards the government or disengaged from the community. DsPH also identified the impact of the anti-vaccination movement as a factor.

“*What I found in XX is that that group that are really unlikely to ever come forward and have the vaccine is actually a much larger percentage. We’ve got quite a strong anti-vaccine movement in XX, and the willingness and availability of misinformation is very strong, and very difficult to overcome*.” (P10, CA)

Other reasons included difficulties accessing vaccination sites, along with residents not being registered with a GP, and hesitancy to come forward for a vaccine among people from non-registered migrant populations. DsPH also pointed out that recorded vaccination rates might not include all vaccinated students, as they could only access vaccination data for students who were vaccinated in England. Several participants said that rates were lower among people from ethnic minority backgrounds, again often linked with levels of deprivation, and among younger people, in part due to the way that the vaccine was rolled out, targeting older groups first, as discussed below, and student populations:

“*I don’t think you can get away from the wider determinant picture here. So, thinking about socioeconomic background, thinking about deprivation … very highly educated who will push their way to the front have been able to take advantage of what’s been on offer really. Whether it’s either testing, understanding about staying at home and social distancing measures that we had, the non-pharmaceutical interventions, or whether it was pushing ahead to get the vaccine done*. ” (P17, AEP)

### Ethnicity and age

When asked about the risk factors for COVID-19 transmission, most participants mentioned the intersectionality of factors such as ethnicity with other factors such as housing or vaccination uptake, although many reported that the role of ethnicity was quite complex. Several participants suggested that when deprivation is accounted for the differences in prevalence by ethnicity might reduce or disappear:

“*There’s an ethnic divide, and deprivation gradients, so different ethnicities are more or less likely to take up vaccination and ….. more deprived populations less likely to be vaccinated, but as deprivation decreases, what we’re seeing…is that that ethnicity gap disappears…So, it’s a function of deprivation and ethnicity, rather than ethnicity per se*.” (P1, CA)

Several participants said that residents from ethnic minority backgrounds were more likely to live in multi-generational households, increasing the risk of COVID-19 transmission as discussed above.

Lack of flexibility in the way that the national vaccination programme was rolled out was reported as challenging, as people in older age groups received the vaccine first, and local authorities could not start vaccinating people in younger age groups until all the older groups had been offered the vaccine nationally. Participants in the AEP were slightly more likely to refer to this, and to report that their area had a younger age profile than average, therefore this issue had had a greater impact on their local authority area than on neighbouring local authority areas with older age profiles:

“*Areas with higher percentages of older people got more vaccination protection, and those of course were areas with the lowest risk…lower historic case rates got higher levels of protection through the vaccination programme, it was a structural inequality*.” (P18, AEP)

Several participants also said that younger people were at higher risk of COVID-19 transmission. They suggested that young adults including students were more likely to work in public facing jobs, including in hospitality and were also more likely to attend social gatherings than older adults. Social gatherings around specific events, which included the release of exam results as well as religious festivals and sporting events, were also linked to higher transmission rates:

“*They [young adults] were obviously much more likely to…be doing the jobs where they’d be at risk, so working as waitresses, waiters, bar staff, et cetera, and also more likely to be engaging in the circulation, the gatherings that were allowed …. There was a very stark decline when our population reduced, and students went home, but actually that age group remained our highest age group …. And of late we’ve seen our post A level results, our 17- and 18-year-olds became our highest rates, again just entirely linked to the hospitality settings, so gathering. I think there’s a really strong connection between people engaging in those social activities where they’re more likely to get close to another, less likely to be wearing face coverings, more likely to be in an enclosed indoor space that seems to be contributing to that*. ” (P10, CA)

### The combined impact of factors that lead to enduring prevalence

Almost all participants said that enduring prevalence was likely to be caused by the interaction of several risk factors, including deprivation, factors related to employment including inability to self-isolate, and factors related to housing including living in overcrowded housing. These factors also intersect with demographic factors such as age, gender, and ethnicity. Although it might be possible that one factor, such as being in an area of multi-generational households, would not lead to enduring prevalence, it was likely that a combination of these factors could. Participants in both the AEP and CA described geographical areas within their local authorities (wards or super-output areas) where they felt that the mixture of several risk factors had combined to cause enduring prevalence, although participants from AEP made slightly more references to this:

*“So, it’s almost as if you could have high levels of variation around ethnicity, and not be an area of enduring transmission. You could be an area of multi-generational households…You may have housing stock that is not as good as some areas… or even income levels could be lower …the issue is, when you start to layer these factors on top of each other. So, enduring transmission comes about by areas where it’s almost the straw that breaks the camels’ back. So, you might have three and be fine, but you won’t have six and be fine, you know*? ” (P5, AEP)

### The similarities and differences between two sets of statistical neighbours

The differences and similarities within and between the two sets of statistical neighbours (two AEP and two CA) were similar to the interview findings overall. Participants who described their local authority area as more deprived than the national average, including the two AEP and one CA, were more likely to identify structural factors such as deprivation, employment and housing as risk factors for transmission. Participants in the CA were more likely to identify mobility as a risk factor. Participants in all four local authorities again asserted that the combined impact of a range of risk factors was likely to lead to high prevalence rates.

### Comparison of the first set of statistical neighbours

There were more similarities than differences between the first set of statistical neighbours, one AEP and one CA. Both participants asserted that their local authority areas were more deprived than the national average and identified links between deprivation and high transmission rates. They identified factors related to employment including lack of sick pay, being in insecure employment or on zero hours contracts, or being self-employed but not qualifying for support grants, which had an impact on residents’ ability to self-isolate if needed, as risk factors for high transmission rates:

*“If you’re a taxi driver, sole income, you don’t qualify for support grants because of the nature of what you do, you get a positive COVID test, you’re in a real dilemma as to whether you’re going to comply, or whether you’re not going to comply, because it then becomes, will I have enough money to keep me going? ...But it’s not wealthy, you don’t have those reserves, you don’t have those opportunities to work from home, like many other areas in the country.* ” (P5, AEP)

Participants in both the AEP and the CA said that the proportion of residents from ethnic minority groups was higher than the national average. They also both felt that residents who lived in larger or multi-generational households had higher transmission risks, and that residents from ethnic minority groups were more likely to live in these households. The AEP participant also emphasised residents’ broader definitions of a household as a risk factor for higher transmission rates. As shown in the quote below, residents in more deprived communities or from ethnic minority groups were more likely to rely on informal networks for a range of issues including child-care, and to view people in these networks, who might live in a different house, as part of their household:

*“So, what we have is, one person in the house gets it, and particularly if you’ve got a multi-generational household, or a wider household, or even informal networks... and clearly, so if one person gets it there, the likelihood is that’s going to be transmitted somewhere else*.” (P5, AEP)

*“If you live in a multigenerational household and you provide the care to other people in your household it’s very difficult for you to isolate effectively. Measures we would put in place, such as offering people hotel stays, for example, so that they could go and isolate somewhere else, were not taken up because it didn’t match with people’s life needs, for example, caring responsibilities, whether it’s children or whether it’s older relatives*.” (P10, CA)

In the interviews, participants in the two local authority areas described similar age profiles, apart from a higher proportion of 16–24-year-olds than the national average identified by the DsPH in the CA. The participant identified this high proportion of 16–24-year-olds, along with the impact of a large student population, as risk factors for high transmission rates. As discussed in the previous section comparing AEPs and CAs overall, young adults, including students, were more likely to work in public facing jobs, including in hospitality. As well as contributing to higher levels of mobility in and out of the area, students and other young adults, were also more likely to attend social gatherings than older adults, and higher transmission rates were also linked to increases in social gatherings around specific events, such as when exam results were released. Participants in both areas identified lower vaccination rates than the national average as a risk factor for high transmission rates. Participants identified a range of reasons for lower vaccination rates, which were similar across the areas, including competing priorities such as work commitments or caring responsibilities, difficulties accessing vaccination sites, difficulties accessing the vaccine due to not being registered with a GP, hesitancy due to cultural or faith beliefs.

“ *And I think there’s this understanding, that, what I’ve seen very clearly, is people want to do the right thing. They genuinely do, but it is not always as easy for people to do the right thing in my city, as it might be in some other areas of the country.”* (P5, AEP)

The DsPH of the CA identified some additional reasons for low uptake, including the impact of the anti-vaccination movement, and also suggested that recorded vaccination rates might not include all vaccinated students, as DsPH could only access vaccination data for students who were vaccinated in England.

*“What I found in XX is that that group that are really unlikely to ever come forward and have the vaccine is actually a much larger percentage.…and the willingness and availability of misinformation is very strong, and very difficult to overcome.” (P10, CA)*

### Comparison of the second set of statistical neighbours

The participant in the AEP asserted that their local authority area was more deprived than the national average and identified links between deprivation and high transmission rates. The CA participant described a more affluent area, with lower deprivation rates than the national average, but identified certain more deprived wards or geographical areas within the local authority with higher transmission rates. The AEP participant also emphasised factors related to employment to a far greater extent than the CA participant, including lack of sick pay and being in insecure employment or on zero hours contracts, which had an impact on residents’ ability to self-isolate if needed.

“ [*We’ve got] the affluent on one side and the most deprived in the south of the borough and we don’t really have any manufacturing, there aren’t big...any offices or anything like that, it’s a very diverse borough.* ” (P9, CA)

“*We had a population who was really trying their very hardest to do everything we were asking them to do. But if you have a zero hours contract and you’re being asked not to go to work, and if you don’t go to work, you don’t bring any money in, you can’t feed your family, that’s incredibly hard for people.* ” (P17, AEP)

Participants in both local authorities said that the proportion of residents from ethnic minority groups was higher than the national average. Both discussed having diverse populations including members of traveller communities. They also both felt that residents who lived in larger or multi-generational households had higher transmission risks, and that residents from ethnic minority groups were more likely to live in these households.

*“I think in our borough mainly we have got very multigenerational households. So, they’re large houses where generation of families live and when we looked at the data most of the transmission was happening within households or within neighbourhoods. So, it’s not...we didn’t find a lot in workplace or outside, it was mainly in families I think that was the biggest challenge for us...I don’t think people really thought seriously about isolating when they had a positive in their own homes.* ” (P9, CA)

Participants in both local authority areas described similar age profiles in the interviews. They both identified lower vaccination rates than the national average as a risk factor for high transmission rates, identifying a range of reasons for this, which were similar across both areas and included competing priorities such as work commitments or caring responsibilities, difficulties accessing vaccination sites, difficulties accessing the vaccine due to not being registered with a GP and hesitancy due to cultural or faith beliefs, although participants reported that they had implemented a range of mitigation strategies to address these issues. In common with other CAs, the participant identified mobility in and out of the local authority area as a risk factor for transmission:

“*I think for us the challenge was because XX traditionally has got really good schools, high achieving schools. So we do tend to get people coming from elsewhere across XX to our schools. ”* (P9, CA)

In alignment with the wider comparison of AEPs and CAs, and the comparison of the first set of statistical neighbours, participants from both the AEP and CA asserted that the combined impact of a range of risk factors was likely to lead to high prevalence rates:

“*In XX it was particularly people from Eastern Europe or people most likely to be doing the frontline work which put them at greater risk. Taxi drivers, bus drivers, healthcare workers, social care workers, and they came from our more deprived communities where they were living in environments where they were least able to follow some of the other things that we were asking them to do*. ” (P17, AEP)

In conclusion, there were many similarities among participants from the two sets of statistical neighbours, both in terms of their perceptions of drivers of prevalence, and their perceptions of and barriers and facilitators to implementing control measures. The two AEP participants, and one of the CA participants described their local authority area as more deprived than the national average and were more likely to identify structural factors such as deprivation, employment and housing as risk factors for transmission. Participants in the CA were more likely to identify mobility between local authority areas as a risk factor.

### Strategies

The strategies that DsPH employed to reduce transmission rates in their local authorities as well as the facilitators and barriers to their local response are described below. Participants discussed taking a lead in local outbreak control by restructuring and reprioritising their pre-pandemic teams at the beginning of the pandemic and creating a shared infection prevention and control strategy for their local authorities. They worked closely with partners (e.g., clinical commissioning groups, social care, primary care) and regional networks (DsPH regional meetings, PHE - now UKHSA) to facilitate a system wide approach to transmission control. Participants discussed a variety of effective mitigation strategies they implemented over the course of the pandemic including local contact tracing, local testing and vaccination efforts, isolation support, communication campaigns, engagement with business and education, and community sectors. Most DsPH made use of most strategies but tailored them as deemed appropriate for local needs.

Other than differences in structural barriers such as variation in levels of deprivation, no major differences in facilitators or barriers to COVID-19 control measures were identified between areas of varying prevalence. Therefore, the facilitators and barriers will be presented in the form of the main themes across AEP and CA combined.

### Facilitators and strategies for reducing transmission

#### Local level facilitators

All DsPH discussed the importance of organising a COVID-19 response at the local level, allowing local control of virus transmission and provision of tailored support for residents in the local authority. For example, the introduction of a locally organised test and trace system was described as an effective approach, in terms of a higher proportion of residents being engaged with the service, to control community transmission while simultaneously offering appropriate welfare support for residents where required:

“*We fundamentally turned the whole of the local authority into a COVID-19 response unit. […] So, we pulled people from our sports services, we pulled people from our customer services across the organisation, to do some of that ground response, to do that follow up in terms of contact tracing, to create, you know, funds were put in place for us to say, well this is the day job*. ” (P5, AEP).

Access to local data, both in terms of the existence of data and ability to access it, was seen by most participants as critical to effective local virus transmission control. This included developing granular data at the local level which gave detailed information of transmission rates according to locations (e.g., wards, postcodes) and population characteristics (e.g., age band, occupation). Also, some participants found the “soft intelligence” (e.g., on cultural differences, attitudes), from knowing local communities and meeting with residents, important in gaining an understanding of influences on transmission rate trends. It provided some additional explanatory value and nuance to objective data. Data were used to direct efforts to control the spread in areas of high prevalence and to tailor messaging and interventions:

*“It’s been the soft intelligence, the local knowledge, and the engagement that we’ve done, as I said, with all of the different sectors and population groups that actually given us the insights that we needed to bend the trends*. ” (P8, AEP).

Many DsPH emphasised the crucial role of consistent, continuous and clear communication in conveying health messages and guidance to the public, including debunking any concerns around testing or vaccination and to promote social cohesion among residents within the local authority. Most respondents engaged with local and social media and identified key individuals in the community, including those in educational, business, or religious settings, to help send out information locally. One of the key facilitators of an effective local COVID-19 response that all DsPH discussed was the close engagement with communities, which allowed them to better understand their communities’ needs and concerns. Direct feedback from communities often fed into the tailored interventions used over the course of the pandemic.

*“So, we would be giving messages, but equally they’d be giving us messages, and that meant that we were able to tailor our responses in a way that really meant that we had a community focus. It was a real coproduction. And that came from us starting that as a principle of this is all of us in it together. So, this was not us just giving them information, it started as a two-way relationship and really developed into a very mature relationship.”* (P10, CA)

Many participants described using various routes of engagement (e.g., door-knocking, social media, organised meetings with local community groups, schools, and businesses) to encourage compliance with COVID-19-related guidance, testing and vaccination uptake. Participants discussed a range of initiatives to make testing or vaccination easier and more convenient for local communities, including providing local vaccination sites in a range of locations such as supermarkets, religious settings, and workplaces:

“*So, part of our strategy was to put vaccination clinics in some of the factories because the theory being, somebody’s come to work, and you’ve got a bit of a captive audience there. And we got loads of people that way, because they wouldn’t have gone, they wouldn’t have made an appointment, but they were at work, and we were sticking it in their arm while they were having their morning break, and that worked.… We put loads of pops ups, drop ins…we did it in mosques, we did it in supermarkets, we did it in community centres and workplaces… having to book it via the national system, was only ever going to work for a certain group of the population really.”* (P2, AEP)

Using a local phone number (which meant that residents were more likely to answer the phone) for contact tracing and having community staff going door-to-door to engage with people were often described as being more effective strategies than more distal methods of engagement (e.g., national contact tracing). Many participants described working with key people in the local area, such as community champions, faith and community leaders, who could act as a gateway to different population groups. Local leaders’ knowledge of their community also helped DsPH to gain better understanding of which COVID-19-related interventions may or may not work in certain communities. Some DsPH were able to build on existing trusting relations with communities to implement interventions and reach vulnerable groups (e.g., people who were homeless, members of traveller communities), and some participants reported that new ways of working had been established between public health teams and community and voluntary groups and organisations during the pandemic. When health professionals had gained the trust of members of these communities they could then work together to improve health outcomes for members of those communities, who might be more likely to seek support with other health issues or to register with a GP:

*“I think it’s the trust. So now what has happened is because they have got a trusted professional, they now want to ask about other health issues…smoking, pregnancy…that’s why we’re linking with the CCG, to make sure they’re registered with a GP and get other healthcare*.” (P9, CA)

The close cooperation between DsPH and partners in public health, business, education, health, and social care was described as important in organising an effective regional and local response during the pandemic. Trusting partnerships helped DsPH to coordinate the testing and vaccination programme and to control virus transmission more effectively within high-risk settings such as schools or care homes. There was also a strong sense of cross-regional learning and support between DsPH from different areas. Many participants engaged regularly with other DsPH and PHE (now UKHSA) as part of the regional public health network to share local knowledge and practices that have been effective in reducing transmission rates.

#### National Level facilitators

Some DsPH acknowledged the necessity of a national level COVID-19 response for managing transmission locally as this helped to reduce transmission rates across the country through testing and lockdown measures and could yield more coherent guidance. The national strategies for test and trace, financial support for isolation and the national vaccination programme were seen as important in facilitating local management of transmission control and welfare support. However, many participants commented on the misalignment between national and local response, which is discussed in the barriers section below and was believed to hinder the effective consolidation of national and local strategies. Participants discussed the effectiveness of national lockdown measures in rapidly reducing transmission rates which the prevalence data has supported. However, there were varying views on what level of lockdown was most effective, with the tier system (activated in Autumn 2020) [16] being seen as not having had the desired effect on transmission rate change as anticipated:

*“It was almost… you needed to get into Tier Three to have an impact. And that did work, so I think there was something about, you know, what was the tiering process trying to achieve. And then you just got local authorities, some wanted to go up a tier, because they wanted to stop things. Some wanted to stay down because they wanted to keep stuff open. And what you saw really, was just it spread region to region. So I think the national lockdown was much more effective in that way.”* (P3, CA)

### Barriers to reducing transmission

#### Local barriers

DsPH mentioned barriers associated with designing effective communications for their communities. People may not be able to understand or may misinterpret guidance put out by local authorities. For instance, participants discussed the need to tailor messages for people with different levels of health literacy and language barriers in diverse communities. The various changes to rules and guidance -one participant (P4, AEP) noted that there had been around 280 changes to national guidance within a year by July 2021- may have also exacerbated the issue of a lack of consistent, clear messaging. Some participants also discussed the issue of stigmatisation of certain population groups which were blamed for high transmission rates. This made tailored interventions and communications directed towards specific communities very challenging and required careful consideration to circumvent additional stigmatisation.

*“You feel a huge responsibility on what you share because of the people that will misuse that information, when people fail to appreciate that it’s the systemic inequalities that exist in society that have put us into this position and that have made these communities experience significant impact of COVID-19.* ” (P2, AEP)

People’s hesitancy to get tested, vaccinated or to self-isolate were mentioned as common barriers to reducing transmission locally. As discussed, hesitancy to get tested or to self-isolate was often described as being the result of competing priorities such as financial barriers or caring responsibilities. Participants in the AEP, which were more deprived overall, emphasised the importance of structural barriers such as financial to a greater extent than those in CA. Some participants also discussed the role of a lack of trust in national government and the systems developed to deal with COVID-19 (e.g. contact tracing, registering COVID-19 test results) as potential drivers for lower compliance with guidance. Other barriers to testing included inaccessibility of testing sites, issues around registering test results and limitations of lateral flow tests, which several participants suggested were not as accurate as Polymerase Chain Reaction (PCR) tests. Vaccination uptake was often discussed by participants to be lower in the younger population and specific population groups. Reasons for hesitancy to get vaccinated, according to DsPH, included attitudes towards vaccination, misplaced concerns about side-effects (e.g., fertility) or inaccessibility of vaccination sites. Some participants argued political motives or mistrust towards government were behind the vaccination hesitancy in young populations, and in certain ethnic and more deprived groups, whilst vaccination inequalities in certain ethnic groups were also often attributed to variances in deprivation levels. Vaccination inequalities in deprived areas were also described as being a common pattern with other types of vaccines, such as the influenza vaccine:

*“Yeah, that’s the other big one, in terms of national policy conflicting with local. So, we’ve actually had a really good vaccine delivery programme in XX, through the primary care networks. And some of the messaging coming around the national vaccination sites, confused the population. So, we had a national vaccination site over the border…that did quite a lot of vaccines for XX. But it wasn’t that accessible for some of our poorer communities and those without cars. But they were getting the messages from the national site before they got the messages from the GP practice. ”* (P3, CA)

The restrictions around data sharing and delays in accessing data in the early stages of the pandemic were often described by participants as a key barrier to their local transmission control. Data sharing restrictions (e.g., NHS data not shared with DsPH) meant that case numbers and data on demographics or location could not be accessed until summer 2020. Some participants reported that, at the time of data collection, there were still some data access restrictions related to, among others, vaccination status of cases, hospitalised patients, or students. This data would have been useful to help gain a complete picture of prevalence rates in local authorities as well as for tailoring local level mitigation efforts to slow the spread of COVID-19. For example, information on correlations between vaccination and hospitalisation could be used for messaging to encourage people to get vaccinated.

*“It was data sharing that’s been the problem, rather than the systems and processes, if you like, because I think that had we been able to get to the data, the systems and processes were there to support whatever action needed to be taken. It was getting the data that was the problem.”* (P2, AEP)

The test and trace system was criticised by some of the DsPH for its lack of sophistication and limited scientific evidence (e.g., the introduction of daily contact testing in schools). Many participants emphasised the uncertainty around the effectiveness of mitigation measures and lack of evidence for causation.

“*So, we haven’t evaluated, so they’re just in the throes of an acute response, there’s just no time or energy to do detailed evaluations. So, we genuinely don’t know what’s been most or least effective, and actually it probably…the answer is that there’s no single thing that has been most or least effective in terms of local strategies. It’s that, kind of, Swiss cheese model where there’s a whole bunch of protective interventions that are needed, there’s no single thing that has done the trick, and then if the collective of all of them together that have then blunted what could have been much worse.”* (P4, AEP)

Participants reported that systematic inequalities and deprivation levels in local authorities, which were higher in the AEP, not only played a crucial part in driving prevalence rates but also hindered their efforts to reduce transmission locally. Families with low income and precarious jobs struggle to cope financially with long self-isolation periods and may also have been more likely to live in poorer housing conditions which makes it more difficult to self-isolate from the rest of the household. Thus, local level interventions to reduce transmission rates were limited by structural, systematic inequalities which were difficult to resolve with local level resources in the short term. Despite having some capacity to provide additional financial and welfare support for residents, DsPH reported that these measures have not been sufficient to provide adequate financial security for communities in deprived areas.

### National level barriers to reducing transmission

DsPH discussed the centralised national approach to managing COVID-19 as one of the key barriers to effective transmission reduction at a local level. Any local deviations of transmission rates from the national level created difficulties for DsPH adequately managing transmission in their local authority. One example was the tier system (activated in Autumn 2020) [15] which placed certain local authorities in local lockdowns without consulting local experts on its feasibility. Some participants talked about the need for flexibility in response to COVID-19, which would allow them to draw on local knowledge to react to prevalence changes, to adapt interventions at the local level as needed, and to provide tailored support for communities.

*“I think the national approach has been a bit one size fits all, and that’s not worked particularly well for us…, I think there was some of the early guidance on…how to self-isolate within your own household, you know, it talked about staying in your bedroom and using a separate bathroom, and that doesn’t apply when you’ve got a multigenerational family living in a terraced house*.” (P6, AEP)

A further issue was the transparency and timing of guidance which was not always deemed appropriate given local circumstances (e.g., locally higher prevalence rates) and not communicated to DsPH and other local stakeholders in a timely manner. Participants stressed that they were sympathetic to the fact that the government faced an unprecedented task to organise a national COVID-19 response and that it needed time to evolve. However, many also expressed the view that multiple changes, inconsistencies and lack of clarity in national messaging and guidance, which was sometimes implemented without consulting them, created challenges for their locally organised communication.

*“Timeliness has been important. The decisions and the updates given at national briefings were never done with the guidance, the regs, all at the same time. You waited after an announcement, for guidance, which means you had a nightmare period of managing millions of questions, without knowing any of the answers. That’s really difficult to deal with locally.”* (P2, AEP)

## Discussion

DsPH, who played a vital role in the local response to COVID-19, identified a number of contributing factors to enduring COVID-19 prevalence and described implementing a range of mitigating strategies during the course of the pandemic.

In the AEP, participants were more likely to identify structural factors such as deprivation, overcrowded housing and low paid or precarious employment as contributing factors to enduring prevalence. These findings align with the findings of a wider project which was conducted by the authors which suggested that structural factors including levels of deprivation, and overcrowded housing were higher in AEP than in CA [12]. They also align with previous research that suggest that large household size and living in a deprived neighbourhood were associated with increased prevalence at times during the pandemic [17]. Because the CA were chosen to match the AEP, many of them also had higher levels of deprivation than the national average, and the DsPH in these areas discussed similar issues related to deprivation. In addition, the current research suggests that differences in infection rates varied during the time period used by SAGE to identify AEPs. Analysis conducted by the Office for National Statistics (ONS) reported that differences in transmission rates between the AEP and CA varied during the course of the pandemic, with many of the CA also reporting higher transmission rates than average at times during the course of the pandemic [14].

Participants also discussed the impact of the intersection of demographic factors, including ethnicity, with other factors such as deprivation. In an exploration of exposure to COVID-19 within residential neighbourhoods, Harris and Brunsdon [18] found that Pakistani, Bangladeshi and Indian groups were disproportionately exposed to the COVID-19 virus in later waves of the pandemic which is likely to reflect the nature of employment of these groups who were more likely to work in public facing roles thereby increasing their risk of exposure.

In both AEP and CA, participants described the impact of low vaccination rates leading to higher prevalence of COVID-19, although participants in AEP were more likely to report that vaccination rates were below the national average than those in CA. Again, this aligns with the findings of a wider study conducted by the authors that suggested that vaccination rates were much lower in the AEP than in the CA, although rates in the CA overall were also below the national average [12]. Participants reported that vaccination hesitancy was of greater concern in deprived areas, including in more deprived wards within their local authorities. They also reported that vaccination rates were lower in communities that showed more mistrust in government, aligning with the findings of previous research demonstrating unequal effects of infection and mortality in more disadvantaged communities [15]. Previous research (e.g. Nafilyan et al.) [5] also demonstrates that occupation is linked to vaccination rates as studies show higher rates in certain occupations (e.g. professional and managerial occupations ) and lower in others, such as people working as general labourers, packers, or cleaners.

The variation in factors such as deprivation, employment, and housing within each local authority area, with DsPH often describing ‘pockets’ of deprivation where a number of risk factors combined, aligns with Daras et al’s research [19] on COVID-19 mortality, which examined the factors that influence prevalence, including ethnicity and overcrowded housing, by ward or super-output area and found high levels of vulnerability clustered within communities. National and local government bodies have also suggested that structural issues linked to age, gender, ethnicity, occupation and geography have exacerbated impacts of COVID-19 on certain communities [20]. Using mortality rates to estimate cumulative infection rates by local authority districts and council areas, Kulu and Dorey [21] reported that, as of June/July 2020, infection rates were positively related to population density of the area and the level of deprivation, which again corresponds with the interview findings.

Bambra et al. [22] describe COVID-19 as a ‘syndemic’: a synergistic pandemic that interacts with and exacerbates a person’s existing non-communicable diseases and social conditions. They suggest that historically, pandemics have been experienced unequally with higher rates of infection and mortality among the most disadvantaged communities. COVID-19 interacts with and exacerbates existing inequalities in determinants of health. Links have been identified, for example, between chronic health conditions and COVID-19 mortality [20]. The research findings align with this, and many participants emphasised that the interaction of various factors (multi-factorial) rather than one single factor contributed to enduring prevalence.

None of the DsPH were confident based on available data exactly how contributing factors interacted and advised that more research was needed to predict enduring prevalence patterns in different areas. The design and nature of this research does not permit further unravelling of the various factors here and highlights the importance of further work in this area to quantify and evaluate the impact of individual and multiple factors that contribute to enduring prevalence.

This research aimed to identify local mitigation strategies to address enduring prevalence rates across different local authorities as well as to explore key facilitators and barriers to the local COVID-19 response. Participants reported that local level interventions to reduce transmission rates were limited by systematic inequalities, which were often related to structural factors such as employment or housing and were difficult to resolve with local level resources in the short term. Other than these structural barriers, no meaningful differences in facilitators and barriers between CA and AEP were identified, perhaps partly due to the fact that the CA were chosen because they were similar to the AEP.

Respondents largely agreed that it was very difficult to determine the effectiveness of strategies for reducing transmission rates and results are primarily based on limited available data and anecdotal evidence. Due to the complexity of transmission risk, there was no single strategy to effectively reduce transmission rates and an array of measures were needed. Local level strategies were generally more focussed on controlling transmission and could not have been able to address or overcome larger, systematic inequalities and deprivation.

The strategies for reducing transmission were similar across local authorities in AEP and in the CA, since many DsPH followed national guidance on transmission control measures (e.g., good practice examples published by the LGA) [9]. DsPH also shared their experiences of public health response strategies with other public health teams to facilitate mutual learning over the course of the pandemic. The strategies to reduce transmission were facilitated by a localised, tailored response, good partnerships with local and regional stakeholders, good data access, consistent messaging, and engagement with local communities. Both the UKHSA [23] and SAGE [24] highlighted the importance of local approaches to provide tailored support for those being disproportionately affected by COVID-19.

A recent government publication outlined a greater role for local public health teams [25]. However, it is crucial this is supported by additional resourcing. The uncertainty around future funding to provide long-term investment in public health was highlighted by the LGA [26] which noted that there will be no real terms increase in public health funding for 2022/23. Some DsPH also voiced concerns over the future of data sharing agreements in post-pandemic times and expressed that preservation of current access to funding and data may be crucial for them to prepare to deal with potential future waves of the pandemic and its long-term effects.

## Limitations of the research

The inclusion of two London boroughs in the comparison group may have skewed the results as they included higher proportions of overcrowded households, homelessness, job, and population density, and people from ethnic minority groups than the national average. Only two CA were statistical neighbours, as defined by PHE [11]. Ideally all the CAs would have been statistical neighbours of the AEP. CAs were chosen because they were similar to AEP, for example in terms of levels of deprivation, and many of them also had higher infection rates than the national average at different times during the course of the pandemic [12, 14]. SAGE defined AEP based on infection rates in the 12 months from 1st March 2020 – if a different time period had been used, it is possible that different AEP would have been identified. This study also suggests that differences in infection rates between the AEP and the CA varied during the time period that SAGE used to identify AEP. Finally, the research is based solely on the perspectives of the DsPH – future research should incorporate a wider range of stakeholders to provide a broader insight into the impact of, and interactions between, different factors.

## Recommendations

This paper recommends that further research is needed on how multiple factors interact in predicting enduring prevalence and which are the most important factors. Further research is also needed in order to be confident about the reasons why differences in prevalence rates changed over time. This might include research on the views and experiences of employers and key health and social care actors, including Directors of Adult Social Care, respondents from community, and voluntary organisations, along with other ‘seldom heard’ groups.

In addition, the body of research on the drivers of COVID-19 is now more extensive than when this research was commissioned and designed in Spring 2021 in response to the SAGE regional differences report [3]. A further recommendation for future research would therefore be to confront DsPH, as well as the other key stakeholders identified above, with the more extensive body of research on the drivers of prevalence, to ask for their opinions on how they feel these drivers contributed to differences in prevalence between AEP and CA.

As part of the interviews, the DsPH were asked what research would be of benefit for them to facilitate an effective local response in the future. Many of them wished to see a better evidence base for local interventions and associated messaging which could be used to shape future interventions. Also, there was consensus that more research was needed to understand more deeply community needs, attitudes, and beliefs with regards to COVID-19 to tailor future messaging and mitigation efforts. Finally, the long-term impact of the pandemic was of interest to the respondents, including effects on individual health, visibility of enduring health inequalities, and the wider system for recovery.

DsPH experiences of the pandemic provide an important opportunity to reflect on effective strategies to respond to future pandemics or health crises. Better alignment of national and local responses may be needed to create consistency and build a system wide approach to reducing transmission, and to ensure that strategies are effective and tailored to local need.

In the shorter term, actions should be taken to tackle modifiable risk factors such as those based on behavioural science, addressing differences in people’s capabilities, opportunities, motivations, and behaviours in response to vaccination and engagement with government guidelines [27]. Access to behavioural sciences resources could be improved, as about half the participants discussed using behavioural science to help inform these initiatives, although most participants talked about relying on national or regional behavioural science resources, with only a minority being able to access resources at a local level. In the longer term, actions to address include house occupancy, housing standards, nature of work, and tackling structural inequalities.

## Conclusion

The research suggests that existing health inequalities influenced the wider picture of prevalence rates of COVID-19. Structural factors including deprivation, employment, and housing, intersecting with demographic factors including ethnicity and age, and vaccination rates, are key drivers of prevalence, and there are key differences in these drivers both within local authorities, and to a lesser extent, between AEP and CA. Further research is needed, ideally at ward or super-output area level, on how these factors combine to predict transmission and how this varies between different areas, and on the relative importance of each of these factors.

Apart from differences in structural barriers in reducing transmission such as levels of deprivation, no major differences in barriers were identified between the AEP and CA. Further research is needed in order to fully explain the differences in infection prevalence between areas and to understand the effectiveness of mitigation strategies.

## Electronic supplementary material

Below is the link to the electronic supplementary material.

Supplementary Material 1

## Data Availability

The datasets generated and/or analysed during the current study are not publicly available due in order to protect participant confidentiality -anonymising the files would require significant redaction, and consent was not sought from the participants to make this qualitative data publicly available but are available from the corresponding author on reasonable request.

## List of abbreviations

ADPH: Association of Directors of Public Health
AEP: Areas of enduring prevalence
CA: Comparison area
DsPH: Directors of Public Health
LAs: Local authorities
LGA: Local Government Association
PHE: Public Health England
PCR: Polymerase Chain Reaction
SAGE: Scientific Advisory Group for Emergencies
UKHSA: UK Health Security Agency
