# Supplementary material for: Areas of enduring COVID-19 prevalence: drivers of prevalence and mitigating strategies
Source: BMC Public Health. 2023 Jun 21;23:1203. doi: 10.1186/s12889-023-15723-7 (PMC10286421; doi:10.1186/s12889-023-15723-7)
Supplement: Supplementary file 1 — Supplementary Material 1 [file 12889_2023_15723_MOESM1_ESM.docx]

Areas of enduring COVID-19 prevalence

Semi-structured interview schedule

The aim of this interview is to help us gain an understanding of why certain places appear to have higher prevalence of COVID-19 infections than others. We would like to gain views from Directors of Public Health (DsPH) and other local stakeholders who can share their knowledge and experience at the local authority (LA) level to discuss potential reasons for enduring COVID-19 prevalence in some regions. We would like to hear your general thoughts on the current situation, what happened in terms of mitigation measures in past waves of the pandemic and what approaches may be taken locally/ regionally to anticipate and reduce areas of enduring prevalence.

Background

1. Can you please tell me a little about your current role and the LA in which you work?

Prompts:

How would you describe your LA in terms of its population/level of deprivation/manufacturing base etc.?

How would you describe the region which your LA is located?

1. How would you describe the changes in COVID-19 prevalence in your LA over the course of the pandemic?

Prompts:

- Rates during the different waves
- How does this compare to neighbouring LAs and LAs with similar characteristics?

We would now like to hear your broad views on potential risk factors for regional enduring prevalence of COVID-19.

1. In your opinion, what are the main factors that contribute to differences in the prevalence of COVID between locations/places?

Prompts:

- What about e.g., population factors, deprivation, nature of work, effectiveness of contact tracing, regional commutes between LAs?
- What is the interplay between the different factors?

1. Why do you think certain areas have sustained high levels of prevalence?

Prompts:

- What and how are these factors driving enduring prevalence?

1. Has the importance/role of these factors changed throughout the timeline of the pandemic? If so, how?

We would now like to hear your broad views on strategies and factors that have been effective in preventing or reducing regional prevalence of COVID-19.

1. How effective do you think national level strategies, policies and guidance are in reducing transmission?

Prompts:

- What are most effective strategies/policies at the national level?
- Has the effectiveness of strategies changed throughout the timeline of the pandemic?

1. Can you tell us about local/regional strategies that have been effective in helping reduce COVID-19 infection rates?

Prompts:

- Examples of strategies/ policies: regulation, guidelines, fiscal measures, environmental/social planning, service provision, legislation, and communication/marketing?
- What have been the most effective prevention strategies for your area/ community?
- Why have interventions been effective/failed? (e.g., different organising of test & trace system)
- What has helped/hindered the introduction of these strategies?
- How effective have these strategies been at different times during the pandemic?
- How is the effectiveness of strategies affected by specific characteristics of localities, places, population, economies?
- What could be done to intervene earlier and curtail prevalence in regions?

1. How have you identified any particular population groups for tailored interventions?

Prompts:

- Is this based on previous data about inequalities or new covid-related data?
- How have you dealt with challenges associated with reaching certain population groups in your interventions?

1. How has the response to COVID-19 been organised locally?

Prompts:

- Which organisations have taken a lead? How have local organisations been working together? E.g., joint forums, with LAs / CCGs / ICSs, resilience hubs?
- Has there been any misalignment/conflict between local, regional, and national strategies/policies/guidance and how have you managed this in your LA?

1. How have LAs been sharing information and learning?

Prompts:

- - Have there been opportunities for you to learn from other DsPH? Are there forums you find useful in exchanging experiences and knowledge?
  - What do you do differently to other LAs? Why?

We are now interested in the data available and its use in decision making and what future research priorities might be.

1. What data, evidence and knowledge is used to inform local decision making?

Prompts:

- What sources of data/information do you find useful?
- How is existing knowledge / data used to inform guidance?
- What are key data or knowledge gaps that need to be addressed?
- How could data be used to anticipate places of enduring prevalence in the future?
- Have there been any issues around data sharing (e.g., between local/regional/national teams)?
- Has behavioural science informed your approach to encouraging hand hygiene, physical distancing, wearing of face coverings, self-isolation, etc? If so, how?

1. What future research do you think would be most useful to provide insights that can support LA practice and decision making?

Prompts:

- Is there anything that would need more research (e.g., patterns/correlations that cannot be explained)?
- What are the key questions for research relating to enduring prevalence and future planning?

1. In your opinion, what are the future challenges for preventing or reducing local / regional enduring COVID-19 prevalence?

Prompts:

- Emergence of new variants of COVID-19
- Support for people to self-isolate (e.g., financial support)
- Impact of vaccination programme
- National strategy for COVID19 transmission management
- Support for places of enduring prevalence in the COVID-19 recovery

1. Are there any other stakeholders you would recommend us contacting to gain a better understanding of disparities in prevalence of covid-19 infection? (names / locations / roles)
2. Finally, is there anything you thought we might discuss, that we have not covered**?**
